# Classifying physical activity levels using Mean Amplitude Deviation in adults using a chest worn accelerometer: validation of the Vivalink ECG Patch

**DOI:** 10.1186/s13102-024-00991-6

**Published:** 2024-10-10

**Authors:** Jim Luckhurst, Cara Hughes, Benjamin Shelley

**Affiliations:** 1 School of Medicine, University of Glasgow, Glasgow, UK; 2 Clinical Research Fellow, Academic Unit of Anaesthesia, Critical Care and Peri-operative Medicine, University of Glasgow, Glasgow, UK; 3 Perioperative Medicine and Critical Care research Group, Department of Cardiothoracic Anaesthesia, University of Glasgow Anaesthesia, Golden Jubilee National Hospital, Glasgow, UK

**Keywords:** Accelerometer, Physical activity, Mean amplitude deviation, MAD, Calibration, Classification, Raw accelerometer data

## Abstract

**Background:**

The development of readily available wearable accelerometers has enabled clinicians to objectively monitor physical activity (PA) remotely in the community, a superior alternative to patient self-reporting measures. Critical to the value of these monitors is the ability to reliably detect when patients are undergoing ambulatory activity. Previous studies have highlighted the strength of using mean amplitude deviation (MAD) as a universal measure for analysing raw accelerometery data and defining cut-points between sedentary and ambulatory activities. Currently however there is little evidence surrounding the use of chest-worn accelerometers which can provide simultaneous monitoring of other physiological parameters such as heart rate (HR), RR intervals, and Respiratory Rate alongside accelerometery data. We aimed to calibrate the accelerometery function within the VivaLink ECG patch to determine the cut-point MAD value for differentiating sedentary and ambulatory activities.

**Methods:**

We recruited healthy volunteers to undergo a randomised series of 9 activities that simulate typical free-living behaviours, while wearing a VivaLink ECG Patch (Campbell, California). MAD values were applied to a Generalised Linear Mixed Model to determine cut-points between sedentary and ambulatory activities. We constructed a Receiver Operating Characteristic (ROC) curve to analyse the sensitivity and specificity of the cut-off MAD value.

**Results:**

Eighteen healthy adults volunteered to the study and mean MAD values were collected for each activity. The optimal MAD cut-point between sedentary and ambulatory activities was 47.73mG. ROC curve analysis revealed an area under the curve of 0.99 (*p* < 0.001) for this value with a sensitivity and specificity of 98% and 100% respectively.

**Conclusion:**

In conclusion, the MAD cut-point determined in our study is very effective at categorising sedentary and ambulatory activities among healthy adults and may be of use in monitoring PA in the community with minimal burden. It will also be useful for future studies aiming to simultaneously monitor PA with other physiological parameters via chest worn accelerometers.

**Supplementary Information:**

The online version contains supplementary material available at 10.1186/s13102-024-00991-6.

## Background

Accelerometers are electrochemical sensors that detect and quantify acceleration of an object in three planes and have become incorporated into a variety of electronic devices, smartphones, and wrist-watches [1]. These devices have enabled remote monitoring of individual’s physical activity levels in the free-living environment over extended periods of time with minimal burden, and thus have developed interest in the fields of sport science, rehabilitation, and many branches of clinical medicine, as they can be applied to a wide range of clinical scenarios and research [2]. The importance of physical activity to health and wellbeing across many physiological systems has been well documented [3] but current methods of measuring these metrics, which rely on self-reporting by patients, suffer the limitations of recollection and response bias and therefore may lack reliability and validity [4, 5]. The rapidly growing market of wearable activity monitors (WAMs) [6], which utilise accelerometers, may offer a cheap and accessible solution by providing an objective measure of physical activity duration and intensity. Recent systematic reviews have already suggested the benefit of utilising WAMs in increasing physical activity in patients with type 2 diabetes [7], cancer [8], and those undergoing cardiac rehabilitation [9], with many other potential clinically significant benefits yet to be explored.

Numerous accelerometery validation studies have been conducted to define specific cut-off points in physical activity intensity levels in adults [10–13] and children [14], however this is predominantly in wrist and hip worn accelerometers such as GENEActive [15] and ActiGraph [16]. Each accelerometer brand and model has a unique algorithm for converting raw accelerometery data into counts per minute and then using cut-points to classify the intensity of the physical activity, moreover, they are placed in different locations of the body. Therefore, direct comparisons of cut-points for physical activity intensity for one brand cannot be generalised to others.

Currently there is a lack of evidence surrounding the use and calibration of chest-worn accelerometers which have several advantages over wrist worn devices. Chest-worn accelerometers are less likely to detect artefactual limb movement and [17] can allow simultaneous monitoring of other physiological parameters such as heart rate variability, heart rate (HR) and respiratory rate (RR) in a single device [18, 19].Moreover, they hold clinical benefits as analysis of the ECG rhythm can allow diagnosis of conditions such as exercise induced arrhythmia.

Recent studies have demonstrated the benefit of using mean amplitude deviation (MAD) in analysing raw acceleration data to define cut-points between sedentary and ambulatory activities in adults [10] and children [14].Sedentary behaviour is typically defined as any waking behaviour done while lying, reclining, sitting, or standing, with no ambulation and ambulatory activity is considered any behaviour involving participant ambulation typically with a slow-paced walk as the lowest accepted activity intensity. Continuous assessment of MAD allows the user’s activity state to be determined at any given point and contemporaneous analysis of physiology during exercise states.

If cut-points in MAD values for defining sedentary and ambulatory behaviour are defined, and these demonstrate high sensitivity and specificity, it will allow further application and study of this accelerometer. Moreover, it will be useful for clinicians aiming to monitor HR, RR, or other parameters offered by the ECG Patch, remotely in free-living conditions in conjunction with accelerometery. The authors for example seek to use this monitor to assess pre-operative fitness by monitoring heart rate recovery in the community setting during activities of daily living, but such validation work is essential and will be useful to other investigators in a range of clinical and non-clinicals scenarios.

The VivaLink ECG patch is a chest worn consumer-orientated accelerometer and is applied to the user’s chest with an adhesive strip and can provide simultaneous monitoring of many physiological parameters such as, HR, RR, body temperature, and HR variability. In this study we aim to calibrate the VivaLink ECG Patch (Campbell, USA) [18] accelerometer and define cut-points in accelerometery obtained from healthy volunteers during activities of daily living data so be able to identify when the wearer is ambulatory or sedentary.

## Methods

### Participants

Participants were recruited by convenience sampling from a publicly distributed email around the study centre. Participants provided informed consent to participate in the study which was approved by the University of Glasgow Medicine, Veterinary and Life Sciences Ethics committee (reference number: 200220144).

### Accelerometer

We used the VivaLink ECG Patch VV330 (VivaLink, Campbell, USA), a chest worn HR monitor with a built-in tri-axial accelerometer. The ECG Patch was applied to each participant under the guidance of the investigator to ensure consistency in the device’s placement (Image 1) according to the manufacturer’s instructions.

### Procedures

Baseline demographic data was collected from each participant who then undertook a series of supervised 2-minute physical activities which imitate typical free-living behaviours. We aimed to validate our accelerometer by analysing accelerometery data against these activities in a controlled environment. There were 9 activities in total and the order of the activities was randomised for each participant using a computer-generated sequence on Microsoft Excel (Microsoft Corp., Redmond, WA). The 9 activities were classified as either sedentary or ambulatory. The sedentary activities included: lying supine, sitting, standing and remaining stationary, typing at computer while seated, writing while seated and standing while moving a 1 kg hand weight. The ambulatory activities included: slow paced walking, normal paced walking, and brisk paced walking. For the duration of these activities, participants were instructed not to talk or engage in any other tasks. These procedures are used as a standard in accelerometery validation studies [10, 14]. Walking paces were self-determined by the participants and the researcher timed the duration of each activity using a stopwatch. The time each activity began and ended was recorded to the nearest second.

### Data processing

Raw accelerometer data was collected in milligravitational (mg) units by the ECG Patch. Accelerometer data for each activity for each participant was stored on a work station and the Mean Amplitude Deviation (MAD) for each activity was analysed in 5 s epochs; 5 s epochs have been established as the optimum epoch length in previous studies [10, 20, 21].

MAD is a statistical measure describing the mean distance of the data point about the mean. This allows quantification and interpretation of accelerometer data, and is calculated as follows:

$$MAD = {\textstyle{1 \over 2}}\sum {|{r_i} - \bar r|}$$

where *n* is the number of data points in the epoch, 𝑟_*i*_ is the i^th^ resultant sample from the epoch and 𝑟̅ is the resultant value of the epoch [22].

For each activity the mean MAD and HR values were calculated from between 15 s after the start of the activity and 15 s before the end of the activity, to minimise the effect changing between activities has on movement and HR data. The mean MAD values for each activity for each participant would then be used to determine the optimum cut-off value between sedentary and ambulatory activities.

### Statistics

MAD values are described as mean and standard deviation. To determine the optimum cut-point in the MAD values derived from accelerometer measurements, we employed a Generalised Linear Mixed Model (GLMMs) approach to perform a logistic regression, by incorporating sedentary and ambulatory behaviours as the binary outcome of interest and the MAD values derived from accelerometer measurements as predictors in our model. To assess the performance of the model and to determine the optimal MAD cut-point, we performed Receiver Operating Characteristic (ROC) curve analysis. The optimal ROC cut-off was defined as the value with the highest sum of specificity and sensitivity (Youden’s index).

To investigate the relationship between MAD and HR, an analysis of covariance (ANCOVA) was conducted. This allowed comparison of the means within each participant while considering the effect of continuous covariates.

## Results

**Fig. 1:**
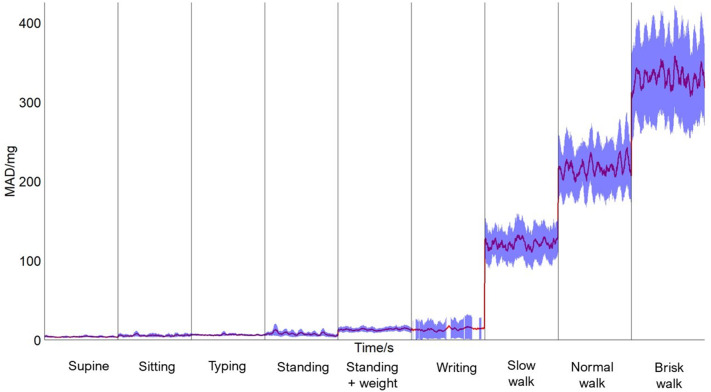
Graph representing MAD versus time for each activity - Mean MAD (expressed in milligravity units (mg)) for each participant versus time for each of the 9 different activities with the 95% confidence intervals. Activities were preformed in a randomised order but have been re-ordered for presentation to aid interpretation

Eighteen healthy adults volunteered to take part in the study: nine men and nine women with a median age of 30.8 (+/- 6.4 years) years, with a mean height, weight and BMI of 173.7 cm (SD 11.9), 76.7 kg (16.3), and 25.3 kg/m^2^ (4.4) respectively. Volunteer demographics are summarised in Table 1; in general, our volunteers reflected a younger and healthier population as recruitment took place in a University Healthcare setting. Table 2 shows the mean MAD value for each subject during each activity and Fig. 1 shows the mean MAD value of all 18 participants for each of the 9 activities over time. Mean MAD values range from 4.1 in the most sedentary activity of lying supine to 319.6 in the most vigorous ambulatory activity of brisk walking. In all cases, ambulatory activities had higher mean MAD values compared to sedentary activities.

**Table 1:** Demographic data of the 18 participants. Data are median (IQR) or n (%) as appropriate. ‘Relevant co-morbidity’ was defined as any condition affecting the cardiovascular system

Characteristic	*N* = 18
Female	9 (50.0%)
Age	30.8 (24.4–37.2)
20–29	7 (38.9%)
30–39	10 (55.6%)
40–49	1 (5.6%)
BMI	24.9 (20.9–27.1)
Relevant co-morbidity	0 (0%)
Self-reported weekly hours of physical activity	5 (3.3–8.5)
Current smoker	2 (11.1%)
Units of alcohol per week	6.5 (1.3–9.5)

**Table 2:** Summary of mean MAD values for each participant across all activities- the mean MAD values (expressed as milligravity units (mg)) for each participant for each of the 9 activities

Sedentary activities							Ambulatory activities		
Participant	Supine	Sitting	Writing	Typing	Standing	Standing+ hand weight	Slow Walking	Normal Walking	Brisk Walking
1	4.2	3.4	5.3	8.6	3.5	4.6	67.8	286.8	475.4
2	2.8	3.4	4.6	8.9	2.5	4.4	114.8	256.5	256.5
3	2.9	2.6	4.6	5.0	2.9	8.7	124.9	237.5	444.6
4	3.4	2.5	11.5	5.8	2.3	7.2	64.8	350.0	456.0
5	3.6	3.6	9.9	3.1	2.8	12.9	132.1	255.6	414.1
6	2.3	2.6	4.9	3.9	3.0	12.8	161.3	290.3	428.9
7	3.9	12.3	7.5	6.9	14.0	20.7	119.9	270.5	396.5
8	7.0	6.5	8.5	6.5	6.6	28.1	123.3	192.6	266.0
9	3.9	5.0	14.1	5.0	11.8	17.8	241.2	301.6	471.1
10	9.0	11.2	6.7	5.6	15.7	24.4	145.0	193.2	262.9
11	2.4	7.2	5.2	8.5	15.9	10.7	148.5	189.3	303.8
12	4.8	4.7	6.1	7.8	12.0	15.6	158.5	285.2	357.4
13	7.2	7.4	6.5	6.2	8.1	10.6	76.2	118.5	156.4
14	3.8	6.3	4.0	7.5	6.1	11.4	111.0	155.9	181.2
15	3.0	11.6	9.7	6.3	3.8	16.0	95.9	128.1	241.0
16	3.1	4.4	7.2	8.3	15.1	14.9	154.9	212.4	270.9
17	2.9	3.5	5.3	7.7	7.9	7.9	70.5	127.3	181.2
18	4.0	3.9	5.1	4.6	5.2	10.1	75.7	120.1	188.3

**Fig. 2:**
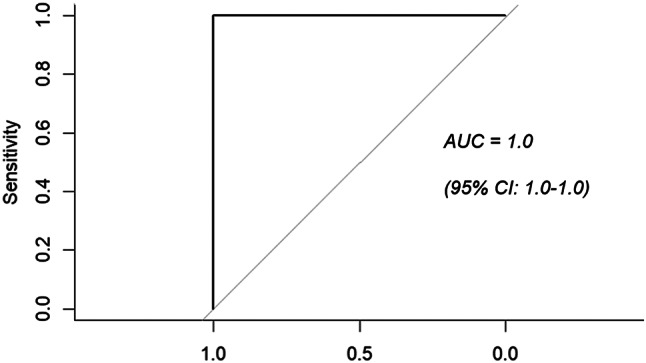
Receiver Operating Characteristic Curve (ROC), analysing the sensitivity and specificity of the MAD cut-point values and the associated area under the curve (AUC) for classifying sedentary and ambulatory activity

The mean MAD value for all sedentary activities was 7.4 compared to the mean MAD value for ambulatory activities of 220.6 (*p* = 0.007). Analysis of ROC curve, (Fig. 2), determined that the optimal cut-point in the MAD values between sedentary and ambulatory behaviour was 47.73 mg. The area under the ROC curve was 0.99 (*p* < 0.001) with a sensitivity and specificity of both 100%.

**Fig. 3:**
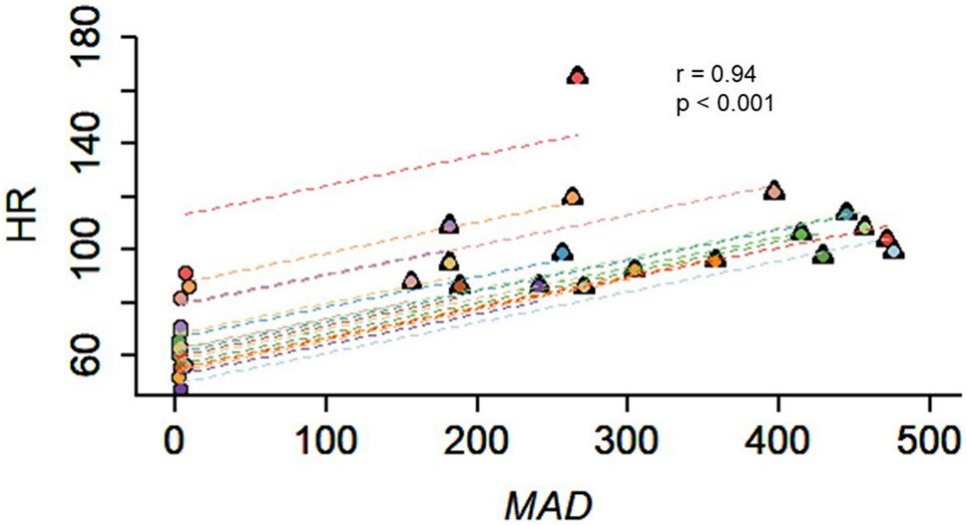
Association between Heart Rate (HR) and Mean Amplitude Deviation (MAD). Each line represents a participant and reflects HR changes as MAD increases. ANCOVA analysis

ANCOVA analysis revealed a correlation coefficient of 0.94 (*p* < 0.001) between HR and MAD values, suggesting a very strong positive correlation between MAD and HR values (Fig. 3). Inspection of Fig. 3 suggests the existence of a consistent linear relationship between MAD and HR.

## Discussion

In this study we calibrated the VivaLink ECG Patch, a chest-worn device with a tri-axial accelerometer, to determine the mean MAD values for various activities of free-living. We successfully recruited an equal number of men and women to the study with an average age and BMI lower than the UK average [23–25], reflecting the recruitment setting being a University Hospital. Visual inspection of our MAD results reveals a clear delineation between sedentary and ambulatory activities with no overlap of the confidence intervals with ambulatory activities averaging a significantly higher MAD value compared to sedentary activities. This is supported by our MAD cut-off value demonstrating a very high sensitivity and specificity allowing us to accurately and precisely discriminate between the intensity of activities users undertake in the community. Moreover, our ANCOVA analysis of HR and MAD supported the validity of our pattern-based intensity classification and aligns with the results we would anticipate with the greater degree of movement resulting in a higher heart rate. This suggests that this accelerometer could be of benefit in remote monitoring of physical activity levels, alongside other physiological metrics irrespective of sex and body morphology of the user. A previous study assessing HR recovery in the community also utilised the VivaLink ECG Patch and demonstrated that participants were comfortable using the device without supervision of an investigator [26]. This may be useful in developing research exploring the benefits of digital health [27], wearable technology [28] and “Hospital at Home” [29]. There are some limitations to this study to consider. Firstly, our volunteers were a sample of healthy, relatively young adults and as such our findings may not therefore be generalisable to adolescents, children or older adults, particular those who may be faced with frailty. However, a previous study found that similar MAD cut-off values were derived for adolescents and adults when using MAD to quantify raw accelerometery and classify activity intensity [14], highlighting the potential utility of our results to a wider population. Secondly, although the activities the participants undertook imitated those of free-living, they were conducted in a laboratory setting under continuous supervision which may have resulted in altered behaviours and influenced both HR and the dynamics of movement. Participants were also instructed not to talk or use phones during these activities, potentially limiting the accuracy of how well they represent activities of daily living.

No formal reproducibility analysis was conducted comparing the results of each activity across participants. However, we had multiple activities classified as either sedentary or ambulatory being analysed against each other for each participant thus demonstrating some reproducibility in our results. Finally, the 9 activities chosen to be investigated represent only a fraction of the number of potential free-living sedentary and ambulatory activities that could be undertaken, for example, driving, cooking, running, swimming or weight-training. If more activities were assessed, there may be sedentary and ambulatory activities which are harder to discriminate purely using a MAD-based cut-point value. However, the activities we chose are frequently used in similar studies [10, 14] and are common examples used by the Sedentary Behaviour Research Network [30].

## Conclusion

The MAD cut-point determined in our study is effective at differentiating between sedentary and ambulatory activities among healthy adults using the VivaLink ECG Patch. This may benefit future studies which utilise chest-worn accelerometers investigating free-living physical activity in conjunction with other physiological parameters.

## Electronic supplementary material

Below is the link to the electronic supplementary material.

Supplementary Material 1

## Data Availability

Data are available on reasonable request - deidentified participant data is available from the corresponding author subject to approval of the sponsor organisation.

## Abbreviations

PA: Physical Activity
HR: Heart Rate
RR: Respiratory rate
MAD: Mean Amplitude Deviation
ROC: Receiver Operating Characteristic
ECG: Electrocardiogram
WAM: Wearable Activity Monitor
mg: Milligravity (the unit for MAD)
RR intervals: The time elapsed between two successive R waves of the QRS signal on the ECG
