# Supplementary material for: Classifying physical activity levels using Mean Amplitude Deviation in adults using a chest worn accelerometer: validation of the Vivalink ECG Patch
Source: BMC Sports Sci Med Rehabil. 2024 Oct 10;16:212. doi: 10.1186/s13102-024-00991-6 (PMC11465818; doi:10.1186/s13102-024-00991-6)
Supplement: Supplementary file 1 — Supplementary Material 1 [file 13102_2024_991_MOESM1_ESM.docx]

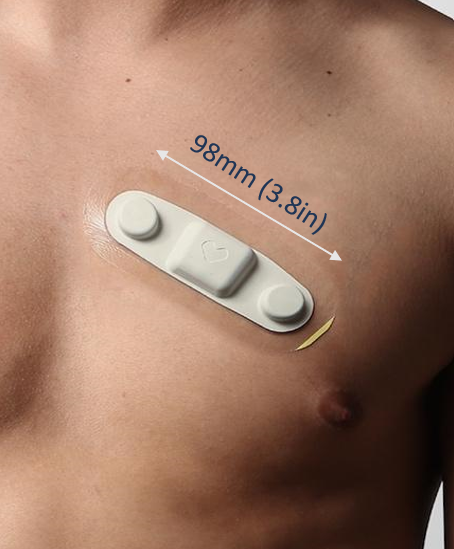


***Image 1:*** *The VivaLink ECG patch fitted correctly on a model’s left chest at an approximately 45-degree angle with the heart symbol pointing up.*

Image 1
